# Taxonomic Profiling of Microbes in Glyphosate-Treated Sediment Microcosms

**DOI:** 10.1128/mra.01183-22

**Published:** 2023-01-04

**Authors:** Madison R. Newman, Darlenys Sanchez, Anna M. Acosta, Bernadette J. Connors

**Affiliations:** a Science Department, Dominican University New York, Orangeburg, New York, USA; Indiana University, Bloomington

## Abstract

Here, we report the impact of glyphosate on bacterial populations in sediment microcosms, determined using 16S amplicon sequencing and shotgun metagenomics with source material from a suburban creek. The 16S amplicon and metagenomic data reveal that members of the genus Pseudomonas are increased by the treatment.

## ANNOUNCEMENT

The herbicide *N*-(phosphonomethyl)glycine, known as glyphosate, is a nonspecific organophosphate herbicide used commonly to kill broadleaf weeds and grasses. Although commonly applied to foliage, measurable quantities of the herbicide have been detected in soil, groundwater, and surface waters (1–4). Several studies have shown that bacterial communities are altered because of glyphosate application (5–8).

Glyphosate microcosms were created with sediment from suburban creek. Sediments were exposed to various amounts of commercial herbicide (0.675% to 2.75% [vol/vol]) for 1 week. DNA was extracted from samples using the ZymoBIOMICS DNA/RNA miniprep kit. PCR was performed based on the Earth Microbiome Project’s 16S rRNA amplification protocol (9). The amplification products were pooled and gel purified on a 2% agarose gel using the Qiagen gel extraction kit. The purified library was quality checked using an Agilent 2100 Bioanalyzer and an Agilent high-sensitivity DNA kit. Sequencing was conducted by Wright Labs (Huntingdon, PA, USA) using Illumina MiSeq v2 chemistry with paired-end 250-bp reads. Forward and reverse demultiplexed paired-end reads were processed using QIIME2 v2021.2 (10). DADA2 (11) was used to generate amplicon sequence variants (ASVs), with the reads trimmed based on Phred quality scores of <15. Taxonomy was assigned using the Greengenes v13.8 database (12).

For shotgun metagenomics, libraries were constructed using the Nextera XT DNA library prep kit from Illumina. The quality of the libraries was determined as described previously. Sequences between 250 and 400 bp were selected via gel purification. The pool was then sequenced at UC Davis Genome Center (Davis, CA, USA), using an Illumina HiSeq 4000 instrument to produce 2 × 150-bp reads. FastQC (13) was used to check the raw data, which were then filtered using fastp (14). Sequences identified as belonging to Homo sapiens were removed using KneadData2 (15). The microbial and functional features of the samples were determined by annotating the paired sequence data using HUMAnN3 (16). RPK (reads assigned per kilobase) normalization was performed using HUMAnN3 during the annotation process. The UniRef90 genes from the functional annotation were mapped to KEGG orthologs (17). Descriptions of all sequencing characteristics are provided in Table 1.

**TABLE 1:** Characteristics and SRA accession numbers of sequences obtained from glyphosate and control sediment samples

Sample name	SRA accession no.	Treatment with glyphosate (%)	Sequence type^*a*^	No. of reads	Relative abundance of Pseudomonas (%)^*b*^
PiermontMarshUnt2M	SRR22193479	Untreated	16S	34,332	0.053
PiermontMarshUnt2A	SRR22193478	Untreated	16S	45,703	0.074
PiermontMarshLoM	SRR22193477	0.675	16S	40,981	0.64
PiermontMarshLoA	SRR22193476	0.675	16S	40,270	0.15
PiermontMarshMedM	SRR22193475	1.375	16S	36,432	2.00
PiermontMarshMedA	SRR22193474	1.375	16S	48,113	1.74
PiermontMarshHiM	SRR22193472	2.75	16S	50,141	9.28
PiermontMarshHiA	SRR22193471	2.75	16S	11,969	1.36
TappanUnt2M	SRR22193458	Untreated	16S	66,301	0.026
TappanUnt2A	SRR22193457	Untreated	16S	62,317	1.36
TappanLoM	SRR22193494	0.675	16S	92,765	4.83
TappanLoA	SRR22193493	0.675	16S	43,101	2.53
TappanMedM	SRR22193492	1.375	16S	67,067	6.57
TappanMedA	SRR22193491	1.375	16S	11,665	16.21
TappanHiM	SRR22193490	2.75	16S	20,293	3.79
TappanHiA	SRR22193489	2.75	16S	18,029	12.72
RockleighUnt2M	SRR22193488	Untreated	16S	39,542	0.11
RockleighUnt2A	SRR22193487	Untreated	16S	43,365	0
RockleighLoM	SRR22193486	0.675	16S	31,783	0.072
RockleighLoA	SRR22193485	0.675	16S	21,324	0.77
RockleighMedM	SRR22193483	1.375	16S	31,095	1.96
RockleighMedA	SRR22193482	1.375	16S	21,414	0.57
RockleighHiM	SRR22193481	2.75	16S	34,743	0.19
RockleighHiA	SRR22193480	2.75	16S	29,505	0.33
MoturisUnt2M	SRR22193496	Untreated	16S	48,809	6.215
MoturisUnt2A	SRR22193495	Untreated	16S	69,827	0.12
MoturisLoM	SRR22193484	0.675	16S	70,491	0.72
MoturisLoA	SRR22193473	0.675	16S	72,277	0.77
MoturisMedM	SRR22193462	1.375	16S	76,593	5.44
MoturisMedA	SRR22193461	1.375	16S	3	NA
MoturisHiM	SRR22193460	2.75	16S	105,557	51.5
MoturisHiA	SRR22193459	2.75	16S	69,569	4.16
SparkillUnt2M	SRR22193470	Untreated	16S	45,738	1.13
SparkillUnt2A	SRR22193469	Untreated	16S	39,689	0.12
SparkillLoM	SRR22193468	0.675	16S	27,811	0.11
SparkillLoA	SRR22193467	0.675	16S	13,643	2.16
SparkillMedM	SRR22193466	1.375	16S	37,711	0.81
SparkillMedA	SRR22193465	1.375	16S	65,204	4.95
SparkillHiM	SRR22193464	2.75	16S	43,978	1.44
SparkillHiA	SRR22193463	2.75	16S	22,753	2.15
Marsh control	SRR22197666	Untreated	MG	32,790,391	0
Marsh treated	SRR22197661	2.75	MG	35,335,201	86.2
Tappan control	SRR22197668	Untreated	MG	34,107,107	0
Tappan treated	SRR22197663	2.75	MG	36,162,594	40.5
Rockleigh control	SRR22197667	Untreated	MG	30,620,071	0
Rockleigh treated	SRR22197662	2.75	MG	40,353,809	1.09
Moturis control	SRR22197669	Untreated	MG	36,371,761	0
Moturis treated	SRR22197664	2.75	MG	50,053,512	56.2
Sparkill control	SRR22197665	Untreated	MG	26,080,480	0
Sparkill treated	SRR22197660	2.75	MG	36,329,037	33.5

a MG, metagenomic.

b NA, not applicable.

Our results revealed that the relative abundance of Pseudomonas increased with glyphosate treatment, regardless of the sample source (Table 1). Through metagenomic analyses, we were able to determine that the Pseudomonas putida group represented the majority of the pseudomonads in the treated samples (16.2% [Tappan], 81% [Marsh], 51% [Moturis], 0.81% [Rockleigh], and 33.5% [Sparkill]). This is in contrast to the relative abundances across all sites and treatments for Enterobacter spp. (2.33% to 3.98%), *Aeromonas* spp. (23.5% to 27.1%), or unclassified sequences (3.21% to 5.12%), which remained comparatively constant. Pseudomonas spp. primarily mapped to the xenobiotic metabolism KEGG pathway in samples treated with glyphosate (56.1% [Tappan], 94.9% [Marsh], 64.8% [Moturis], 2.5% [Rockleigh], and 37.5% [Sparkill]), whereas the unclassified sequences primarily mapped to this KEGG pathway in untreated samples across all samples and sites.

Understanding these changes can help us develop genetic markers that may reveal glyphosate contamination without the need to detect and quantify glyphosate itself, a laborious procedure that is both costly and requires specialized equipment (18).

### Data availability.

The raw sequencing data are available at the NCBI Sequence Read Archive (SRA) under BioProject accession number PRJNA898361. The SRA accession numbers are listed in Table 1.
